# Remdesivir and Ledipasvir among the FDA-Approved Antiviral Drugs Have Potential to Inhibit SARS-CoV-2 Replication

**DOI:** 10.3390/cells10051052

**Published:** 2021-04-29

**Authors:** Rameez Hassan Pirzada, Muhammad Haseeb, Maria Batool, MoonSuk Kim, Sangdun Choi

**Affiliations:** 1 Department of Molecular Science and Technology, Ajou University, Suwon 16499, Korea; rameez_hassan99@yahoo.com (R.H.P.); haseeb3389@hotmail.com (M.H.); mariabatool.28@gmail.com (M.B.); moonskim@ajou.ac.kr (M.K.); 2 S&K Therapeutics, Woncheon Hall 135, Ajou University, Suwon 16499, Korea

**Keywords:** ledipasvir, remdesivir, SARS-CoV-2

## Abstract

The rapid spread of the virus, the surge in the number of deaths, and the unavailability of specific SARS-CoV-2 drugs thus far necessitate the identification of drugs with anti-COVID-19 activity. SARS-CoV-2 enters the host cell and assembles a multisubunit RNA-dependent RNA polymerase (RdRp) complex of viral nonstructural proteins that plays a substantial role in the transcription and replication of the viral genome. Therefore, RdRp is among the most suitable targets in RNA viruses. Our aim was to investigate the FDA approved antiviral drugs having potential to inhibit the viral replication. The methodology adopted was virtual screening and docking of FDA-approved antiviral drugs into the RdRp protein. Top hits were selected and subjected to molecular dynamics simulations to understand the dynamics of RdRp in complex with these drugs. The antiviral activity of the drugs against SARS-CoV-2 was assessed in Vero E6 cells. Notably, both remdesivir (half-maximal effective concentration (EC50) 6.6 μM, 50% cytotoxicity concentration (CC_50_) > 100 µM, selectivity index (SI) = 15) and ledipasvir (EC50 34.6 μM, CC_50_ > 100 µM, SI > 2.9) exerted antiviral action. This study highlights the use of direct-acting antiviral drugs, alone or in combination, for better treatments of COVID-19.

## 1. Introduction

Coronaviruses are enveloped viruses belonging to the *Coronaviridae* family and are notorious for their ability to cause respiratory infections in humans [1]. Coronaviruses are categorized into four subgenera (based on differences in protein sequences) named alpha (α), beta (β), gamma (γ), and delta (δ) [2]. To date, β-coronaviruses have been identified as the causative agents of respiratory infections in humans, including the common cold. In 2019, the newly identified coronavirus SARS-CoV-2 causing COVID-19 caused a global pandemic. The genome of coronaviruses has a size ranging from 27 to 32 kilobase pairs (kbp) and codes for open reading frames 1a and 1b (ORF1a and ORF1b), i.e., polyproteins that regulate viral replication [3,4]. Four other coronavirus strains have been known previously to cause disease in humans and are named 229E, HKU1, NL63, and OC43 [5].

There are different nonstructural proteins (nsps) named nsp1 through nsp16 in coronaviruses that play a major role in genome transcription and replication; however, the exact function of a few nsps remains unidentified. Structural proteins play an important role in viral infection and virion assembly, whereas the spike-like S protein facilitates surface attachment to host cells [5,6]. The M protein with transmembrane domains binds to the nucleocapsid and shapes the virion [7,8]. The envelope protein (E protein) is important for viral infection pathogenesis because it performs a crucial function in virion budding and assembly. The N protein is composed of two domains: one with the ability to bind to the viral genome and a nsp3 triggering the replicase–transcriptase complex and the encapsulation of the viral genome. Most RNA viruses, with the exception of retroviruses, require an RdRp for transcription and replication of their genome; therefore, this protein is crucial for their survival [9]. RdRp is highly conserved among RNA viruses and is composed of a 900-amino-acid catalytic core that consists of the palm, fingers, and thumb domains [10]. RdRp has been identified as a conserved protein within a group of RNA viruses and therefore could be an attractive research object for analyzing the process of nucleic acid synthesis and for the development of antiviral drugs [10,11].

Soon after the SARS-CoV-2 pandemic, many in vitro studies and clinical trials were conducted to identify potent drugs with high antiviral activity. In this regard, favipiravir is one of the first antiviral drugs authorized by the National Medical Products Administration of China for use against SARS-CoV-2 [12]. In the current scenario, the efficacy of the antiviral drugs may not be as effective against virus due to single nucleotide polymorphisms which changes the viral proteins [3]. A similar phenomenon can also be observed in SARS-CoV2, whereby the viral proteins (i.e., spike protein) acquire mutations due to SNPs and consequently escape being targeted by antiviral drugs [13,14,15,16]. However, the conserved protein such as RdRp could be an appropriate target to understand the mechanism of nucleic acid synthesis and the subsequent identification of already available antiviral drugs [17,18,19,20,21]. Several other drugs, such as remdesivir, ribavirin, and sofosbuvir, are currently in different phases of clinical trials for evaluation of their anti-RdRp activity. Nonetheless, a multistep approach is needed to speed up the drug discovery process [12,22,23,24].

In this context, to overcome the problems associated with the discovery of effective antiviral drugs, the current study was conducted to determine potential effectiveness of FDA-approved antiviral drugs against SARS-CoV-2 replication. Here, we employed a combined multistep in vitro and in silico approach, involving virtual screening, docking, and molecular dynamics (MD) simulations to identify a lead compound effective against the virus. The best drug was then selected based on a binding score, stability, and its interaction with key amino acid residues to be confirmed by in vitro tests. The antiviral activity of the top hits (remdesivir, ledipasvir, and paritaprevir) was then assessed in Vero E6 cells. The high antiviral activity of remdesivir (half-maximal effective concentration (EC_50_) = 6.6 μM) and ledipasvir (EC_50_ = 34.6 μM) observed in Vero E6 cells against SARS-CoV-2 highlights their potential utility as an effective therapeutic intervention to be tested in combination to achieve better efficacy for further clinical evaluation. The findings of this study will also help physician scientists to treat COVID-19 patients with an appropriate drug.

## 2. Materials and Methods

### 2.1. Protein Model Preparation and Active-Site Identification

The three-dimensional (3D) structure of the SARS-CoV-2 RdRp protein was retrieved from the Protein Data Bank (PDB; ID: 6M71) [25]. The retrieved cryo-electron microscopy structure was prepared using the Molecular Operating Environment (MOE) software [26]. All water molecules and associated cofactors (nsp7–8) were removed, and hydrogen atoms were added to the structure. All missing atoms were modeled, and energy was minimized to remove any steric clashes and to optimize bond lengths and angles. For energy minimization, the AMBER10: EHT force field was utilized with a 0.01 rms kcal/mol gradient. The structural topology was reviewed for any abnormalities. The active site of RdRp is located in the seven conserved motifs (A to G). The sterol-sensing domain sequence (amino acid residues 759–761, particularly residues K545 and R555) was found to be a hotspot.

### 2.2. Library Preparation

All the available antiviral drugs (*n* = 63) approved by the FDA in the last 50 years were selected for virtual screening, and their 3D structures were downloaded from the PubChem database [27,28]. The antiviral drug library was prepared in MOE, explicit hydrogen atoms were added, and all molecules were subjected to energy minimization by means of the Merck molecular force field 94× (MMFF94×) with a root mean square gradient of 0.1 [29,30].

### 2.3. Virtual Screening and Molecular Docking

Docking was performed using Moe-Dock (Chemical Computing Group Inc., Montreal, QC, Canada). Each FDA-approved antiviral drug was docked separately to the active site of the RdRp protein. At the first step, water molecules were removed, and 3D protonation and energy minimization were performed using the MOE software with the following parameters: force field MMFF94X, gradient 0.05, and current geometry. The FDA-approved antiviral drug library was docked into the catalytic binding site of RdRp (Arg555, Val557, Asp618, Asp623, Thr680, Asn691, Ser759, Asp760, and Asp761). Next, active-site residues were selected, with 10 conformations, and the prepared library was screened against the selected residues. The protein and ligand atoms were kept flexible, and the triangle-matcher placement method was used along with the London dG scoring function. Among the top 10 hits, three drugs were selected (based on a docking score, binding mode, hydrogen bonding, and interactions) for MD simulations; namely, remdesivir, ledipasvir, and paritaprevir were chosen, and their intermolecular interactions were studied by means of PyMOL and MOE [31]. After that, the docked complexes were subjected to MD simulations.

### 2.4. The MD Simulation Protocol

#### 2.4.1. Ligand Topology Generation

The official CHARMM general force field (CGenFF) server was used to build the topology of the selected ligands for the MD simulations [32]. Initially, the ligand molecules were converted into mol2 format, hydrogen atoms were added into the correct tautomeric and protonation state, and the bond order was corrected accordingly. Next, the ligands in the mol2 format were uploaded to the *CGenFF* server to generate a CHARMM-compatible stream file. The stream file contains ligand topological information, such as atom types, charges, and bond parameters. The stream file was then converted into a Gromacs-compatible file using a python script that generates a PDB file, parameters, and topology files for the ligand. The generated ligand molecular structure was then saved in the Gromacs-compatible format.

#### 2.4.2. MD Simulations

The RdRp complexes with the chosen FDA-approved drugs were subjected to MD simulations to discern the dynamics and interaction behavior of these compounds. The all-atom simulation method was used to gain insights by solving Newton’s equation of motion. Initially, SARS-CoV-2 RdRp in complex with a drug (remdesivir, ledipasvir, and paritaprevir) and the apo-RdRp protein (PDB ID: 7BV1) were subjected to energy minimization to remove steric effects and to optimize the structure. The CHARMM36 all-atom force field was utilized [33]. Periodic boundary conditions were applied, and the protein was placed inside a cubic box with 10 Å distance between the boundary and the protein surface. The TIP3P water model was employed to solvate the systems, and the system was neutralized by the addition of Na^+^ ions. The preprocessing steps were then executed, which included energy minimization and temperature and pressure equilibration. Energy minimization was carried out by the steepest-descent algorithm for the subsequent 50,000 steps, followed by NVT and NPT ensemble for 100 ps in order to attain a point of maximum force of 1000 kJ mol^−1^ nm^−1^. Then, the temperature equilibration was performed via the V-rescale algorithm at 300 k with a time constant of 0.1 [34] The particle mesh Ewald algorithm [35] was used to calculate long-range electrostatic interactions, while short-range electrostatic and van der Waals were calculated by specifying 1.2 nm cutoff distance. Additionally, the Parrinello–Rahman algorithm was applied to achieve pressure equilibration at 1 bar. After that, 100 ns (nsteps 50,000,000) MD simulations were carried out for protein–ligand complexes and the apo-RdRp protein using the Gromacs software [36]. The data analyses were performed by means of Gromacs built-in tools, MOE, and PyMOL.

### 2.5. Post-MD Simulation Data Analysis and Visualization

The trajectories obtained throughout the MD simulations were researched by further analyses, such as root mean square deviation (RMSD) computation to measure the system stability, whereas root mean square fluctuation (RMSF) was employed to evaluate flexibility at the amino acid residue level. The radius of gyration (R_g_) was calculated to measure structure compactness. Principal component analysis (PCA) was performed to evaluate functional dynamics of the protein. Additionally, to characterize stable and variant states of the protein, free energy landscape (FEL) analysis was performed. Lastly, to identify correlated motions of residues, a dynamic cross-correlation matrix (DCCM) analysis was performed. For all these analyses, Grace (plasma-gate.weizmann.ac.il/Grace), R packages, and Gromacs built-in and standalone tools were used.

### 2.6. Principal Component Analysis (PCA)

This is a dimensionality reduction method predominantly serving to demonstrate slow and functional motions of biological molecules [37]. To perform PCA, a covariance matrix was calculated by diagonalizing and solving eigenvalues and eigenvectors. The direction of the motion is represented by eigenvectors, whereas the magnitude of the motion and direction is denoted by eigenvalues. Furthermore, the covariance matrix for the illustration of PCA was computed with Gromacs analysis tools g_covar and g_anaeig [38]. Consequently, the trajectories were changed to DCD format by means of stand-alone software Wordom [39]. The PCA was further executed via the Bio3D analysis tool by a method described previously [40].

### 2.7. The Free Energy Landscape (FEL)

An FEL is determined to characterize all possible conformational changes of a protein in MD simulations [41,42]. The FEL represents two variables that reflect a stable and transient state of the protein and was computed here from a probability distribution composed of the first two eigenvectors from the essential plane. Positions of the interacting molecules in the system were characterized by focusing on their respective energy levels [43]. In the calculation of the FEL, protein stability was identified by Gibb’s free energy calculation. Here, we utilized gmx sham, a Gromacs tool, to compute the FEL. In this study, the apo form and protein–drug complexes were evaluated via the following equation:

G_i_ = −K_B_Tln(N_i_/N_max_)

where K_B_ is the Boltzmann constant, T denotes temperature (300 K), N_i_ is the population of bin i, and N_max_ is the population of the most populated bin. A color-coded model depicts various energy levels.

### 2.8. The Dynamic Cross-Correlation Matrix (DCCM)

The DCCM analysis was carried out to identify correlated motions of residues upon drug binding, and the Bio3D package available in the R software was employed to calculate residue–residue dynamic cross-correlations [44]. To build the matrix, only Cα atoms were selected. Covariations of the matrices were calculated on calling “cov2dccm” upon calculating Pearson’s covariance matrix correlation coefficients from the coordinates. Based on the following equation, the cross-correlation ratio and matrix (C_ij_) represent time-correlated data between atoms i and j of a protein [45,46]:

$$C_{\mathrm{ij}}={\left\langle \Delta r_{i}\cdot \Delta r_{j}\rangle ∕\{\langle \Delta r_{i}\rangle 2\langle \Delta r_{i}\rangle 2\right\}}^{1/2}$$

In the above equation, both Δr_i_ and Δr_j_ show average locations of the ith and jth residues, respectively. The angular brackets represent the average time. The positive values indicate correlated motions, and negative values indicate atomic displacements in opposite directions.

### 2.9. MM-PBSA Calculation

The MM-PBSA method was used to calculate the binding free energy between protein and ligand complexes. In total, 100 frames were extracted from the trajectories and the total energy of the system was calculated through the following molecular Mechanic/Poisson–Boltzmann Surface Area (MM-PBSA) equation

ΔG(Binding)= G(Complex)− G(Receptor)− G(Ligand)

In the above equation, G (complex) represents the total free energy of the protein-ligand complex, whereas G (receptor) and G (ligand) are total free energies of the isolated ligand and protein in solvent. Thus, the ‘g_mmpbsa’ tool from the Gromacs package was used to perform MM-PBSA calculations [47]. A detailed explanation of the method used in the calculations can be found in previous studies [48,49,50]

### 2.10. In Vitro Activity of the Antiviral Drugs

The compounds were purchased from MolPort (https://www.molport.com), and 20 µM stock solutions were prepared in dimethyl sulfoxide (50–100 µL). The sequence of a SARS-CoV-2 strain isolated from COVID-19 patients by Korea Disease Control and Prevention Agency was used (BetaCoV/Korea/KCDC03/2020). One-hour post infection, the viral inoculum was removed, and Vero E6 cells were treated with serial dilutions of the candidate drugs in the infection medium. After 48 h, the cells were stained with an antibody against the constituent protein of the virus to evaluate the degree of inhibition of viral replication in the sample, and cell viability (drug toxicity) was assessed by examining the presence of the nucleus in the cells. If a drug was effective in a sample, all the cell nuclei were stained (drug toxicity 0%), and the virus component protein was not stained by the antibody (inhibition 100%). Dilutions of the solvent in the infection medium were utilized to set up mock-treated controls. The virus-inhibitory effect was checked using the antibody against the viral protein as a vehicle, and cell viability (drug toxicity) was evaluated via the nuclear staining of cells.

## 3. Results and Discussion

### 3.1. Virtual Screening and Molecular Docking

**Figure 1:**
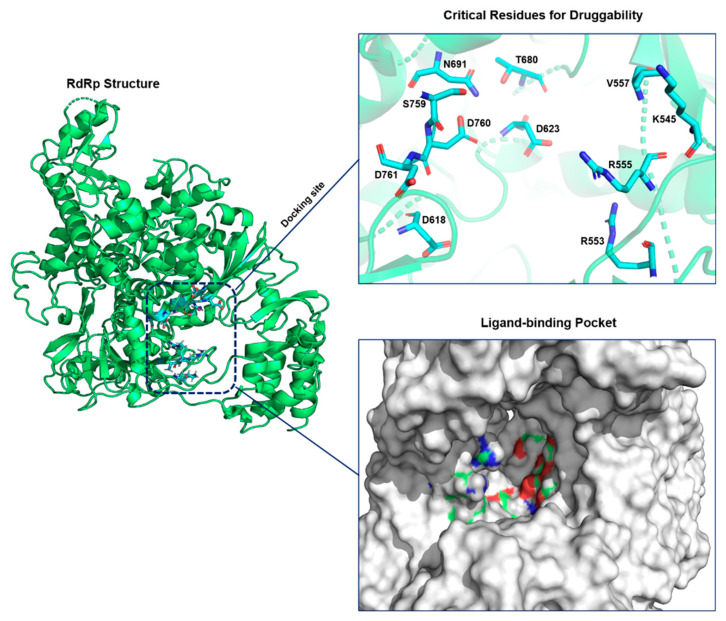
Structure of SARS-CoV-2 RNA-dependent RNA polymerase (RdRp) and its critical amino acid residues. On the right, a zoomed view of the active-site residues is shown in detail. RdRp is highlighted in green, and active-site binding residues are presented as cyan stick models.

In this study, we chose the virtual-screening approach to identify potential antiviral drug candidates against SARS-CoV-2 RdRp that target active-site residues (Figure 1). The active site of RdRp is highly conserved among various microbes [51]. The criteria for selecting the best compounds are the docking score (S) and multiple interactions with the active-site residues. The top 10 compounds that satisfied the filtering criteria were visualized manually. Finally, out of these top hits, three antiviral drugs were selected for comparative analysis.

### 3.2. The Analysis of the Interaction of Top Hits with RdRp

**Figure 2:**
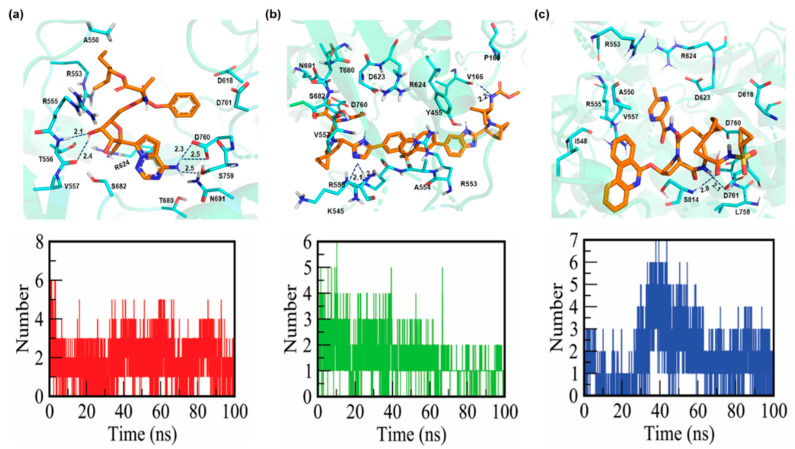
Binding mode and hydrogen bonds of remdesivir, ledipasvir, and paritaprevir with RdRp. Detailed interactions occurring within 4 Å of the ligands are shown. RdRp is lime-green (transparent). Ligands ledipasvir, remdesivir, and paritaprevir are presented as orange stick models. (**a**) Interactions of remdesivir and the number of hydrogen bonds with important residues in the ligand-binding pocket of RdRp. (**b**) Binding mode of ledipasvir and the number of hydrogen bonds with RdRp. (**c**) Binding mode of paritaprevir and the number of hydrogen bonds with RdRp.

The molecular docking of FDA-approved antiviral drugs with the SARS-CoV-2 RdRp crystal structure was performed next. The docking scores of the top hits (remdesivir, ledipasvir, and paritaprevir) from the drug library implied a strong interaction with the key residues. For ledipasvir, the docking score was found to be −8.9 kcal/mol. It forms three hydrogen bonds with active-site residues. The amidine group of R555 engages in two hydrogen-bonding interactions with the imidazole group of ledipasvir, with bond lengths of 2.1 and 2.6 Å. Similarly, the carboxyl group of another amino acid, V166, forms a hydrogen bond with the methoxycarbonyl amino group (bond length of 2.2 Å; Table 1, Figure 2a).

Remdesivir, with a docking score of −8.3 kcal/mol, yielded five hydrogen bonds. The carboxyl group of T556 forms two hydrogen bonds with the oxygen atom of the 5-cyano-3,4-dihydroxyoxolan-2-yl moiety, with bond lengths of 2.1 and 2.4 Å. Similarly, the deprotonated carbocyclic group of D760 and amino group of N691 form two and one hydrogen bonds, respectively, with the hydrogen and nitrogen atoms of the nucleobase substituent 4-aminopyrrolo[2,1-f][1,2,4]triazin-7-yl group, with bond lengths of 2.3 and 2.5 Å (Table 1, Figure 2b).

The docking score of paritaprevir was −9.4 kcal/mol, and this drug participates in hydrogen bonding with the key binding-site residues (D761 and S814). The carboxyl group of D761 forms a hydrogen bond with the amide group of an aza-macrocyclic complex with a bond length of 2.1 Å. Additionally, the amino group of S814 engages in a hydrogen bonding with the carbonyl group attached to the acyl-sulfonamide moiety with a bond length of 2.0 Å (Table 1, Figure 2c). This study suggests that remdesivir engages in stronger interactions with RdRp than ledipasvir and paritaprevir do, thereby inhibiting the template entry site more effectively. It is known that aspartate, serine, and arginine play critical roles in the formation of hydrogen bonds [52]. Furthermore, the number of hydrogen bonds between the protein and ligands is shown in (Figure 2a–c). During MD simulations, the number of formed hydrogen bonds in both RdRp–ledipasvir and RdRp–remdesivir complexes varied between 0 and 6, while for RdRp–paritaprevir, the hydrogen bond number varied between 0 and 7.

### 3.3. Measurement of Variations in the Cα Atoms of RdRp in the Presence and Absence of Drugs

**Figure 3:**
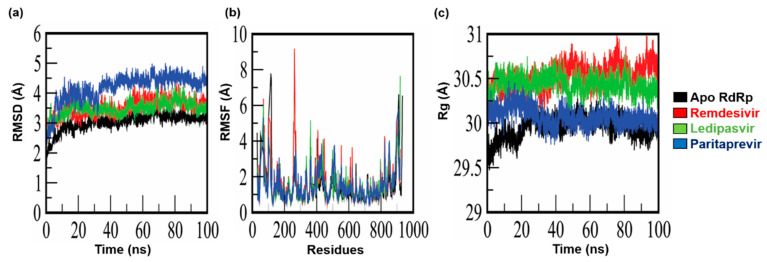
MD simulation results on apo-RdRp and complexes of RdRp with remdesivir, ledipasvir, and paritaprevir. (**a**) Root mean square deviation (RMSD) of the apo-form of RdRp and the complexes; (**b**) root mean square fluctuations (RMSFs) of the apo-form of RdRp and the complexes; (**c**) the radius of gyration (Rg) of the apo-form of RdRp and the complexes.

All the RdRp–ligand complexes were simulated in an aqueous environment for 100 ns each to measure the conformational changes and discern the dynamics of stability. The deviation of backbone atoms was measured by means of RMSD. RMSDs of all the systems are given in Figure 3. To characterize the dynamic behavior of RdRp during the interaction with the selected FDA-approved antiviral drugs (remdesivir, ledipasvir, and paritaprevir), MD simulations of the RdRp-drug complexes, with each selected drug, were performed in an explicit water environment. Dynamic behaviors of the protein and ligands were analyzed individually using MD simulation trajectories. The RMSDs of the complexes were assessed and compared with the RMSD of apo-RdRp (Figure 3a). In this analysis, the RdRp–ledipasvir complex displayed steady incremental deviation during the simulation with RMSD starting from 2.1 Å and reaching 3.3 Å. Similarly, RdRp–remdesivir showed incremental deviation (2 to 3.6 Å) throughout the simulation, along with some acceptable convergence during the time intervals. By contrast, the RdRp–paritaprevir complex manifested a dramatic increase in the deviation from 2 to 4 Å between nanosecond 0 and nanosecond 6, but soon the complex stabilized and stayed stable throughout the rest of the simulation, with the RMSD reaching 4.3 Å. In the comparison, the RMSD of RdRp in the complexes with remdesivir and ledipasvir yielded a trajectory similar to that of the apo-form of RdRp, with slight variation during the time interval. Overall, the RdRp–ligand complexes attained an average RMSD of approximately 3.7 Å by the end of the simulations.

Moreover, we measured residue flexibility by means of RMSF. In the case of apo-RdRp, greater fluctuation primarily occurs in residues K50 (3.099 Å), R116 (7.783 Å), N403 (4.14 Å), and D910 (6.617 Å), which are present in loop regions, while no significant fluctuations were observed in other regions (Figure 3b). Additionally, RMSFs of the active-site residues, R555 (0.876 Å), V166 (1.301 Å), T556 (0.854 Å), D760 (1.157 Å), N691 (0.727 Å), D761 (1.038 Å), and S814 (1.01 Å), indicated no significant fluctuations (Figure 3b).

Nevertheless, for the RdRp–remdesivir complex, significant fluctuations were noted in the loop regions, especially surrounding residues Y69 (6.38 Å), F103 (5.317 Å), and T262 (9.17 Å). On the other hand, the scores (Cα RMSFs) for the catalytic binding-site residues T556, D760, and N691 were 1.512, 1.536, and 0.94 Å, respectively. Additionally, a similar fluctuation pattern was identified in the loop region of the RdRp–ledipasvir complex; in particular, residues Y69, F102, H362, M906, and E919 featured greater fluctuations. The scores (Cα RMSFs) for the active-site residues R555 and V166 were 1.741 and 1.34 Å, respectively.

In the RdRp–paritaprevir complex, loop region residues Y69 (5.307 Å), F102 (4.221 Å), T262 (3.157 Å), R365 (3.156 Å), E431 (3.806 Å), K508 (3.67 Å), D824 (3.992 Å), G852 (4.02 Å), and L907 (5.801 Å) showed greater fluctuation. The scores (Cα RMSFs) for the catalytic binding-site residues D761 and S814 were 1.489 and 1.791 Å, respectively.

Overall, the protein–ligand interactions were found to differently affect residue flexibility and internal dynamics. The average RMSF for the RdRp–drug complexes are 1.49 Å, which is slightly higher than that of unbound RdRp (1.00 Å).

### 3.4. Calculation of Equilibrium Conformation of Systems

Structural compactness of all the systems was measured through the calculation of R_g_ of their MD trajectories, and average values were obtained. The effects of drug binding on R_g_ of the protein were measured and compared with the apo-RdRp protein structure. The average R_g_ of apo-RdRp turned out to be 29.9 Å, and the system remained stable and compact. For the RdRp–ledipasvir complex, the average R_g_ was 30.2 Å and showed negligible deviation in terms of compactness and stability as compared to the apo-structure. Similarly, an average R_g_ of 30.0 Å was observed in the RdRp–paritaprevir complex. On the contrary, for the RdRp–remdesivir complex, R_g_ increased to 30.5 Å (Figure 3c). Thus, RdRp in all the complexes showed sustained stability and compactness corresponding to native RdRp.

### 3.5. Analysis of Essential Dynamics of Protein

**Figure 4:**
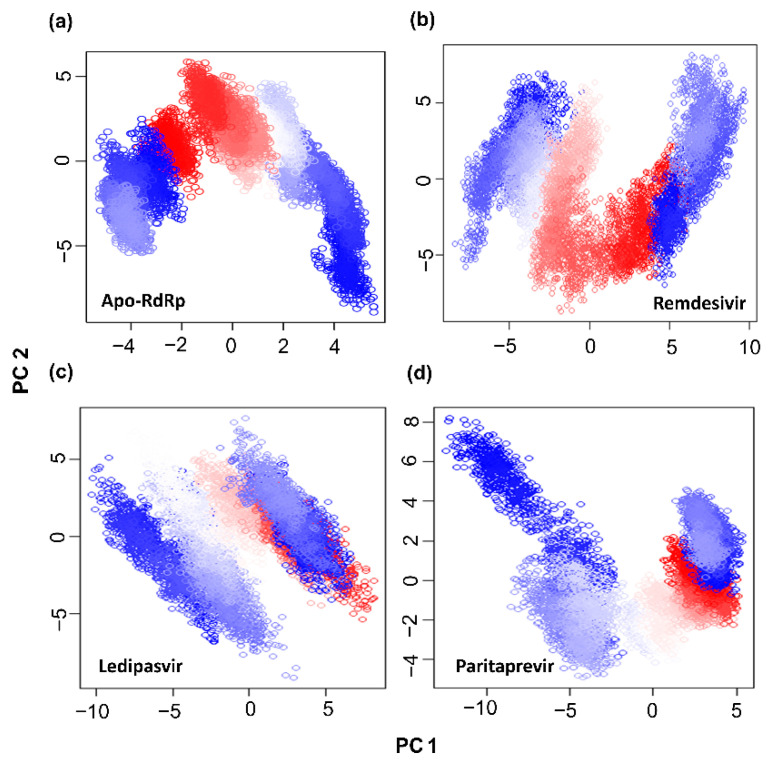
Projection of protein atoms in phase space along the first two principal eigenvectors. (**a**) The apo-RdRp protein; (**b**) the RdRp protein complexed with ledipasvir; (**c**) the RdRp protein complexed with remdesivir; (**d**) the RdRp protein complexed with paritaprevir.

Essential dynamics in a protein are controlled by switching between different conformations, and the phenomena governing this modular nature of the protein are controlled by overall collective motions [53]. This concept is important in many biological processes and plays an important role in biological signaling pathways. In this context, considerable flexibility and rigidity are required for a protein to be functional, especially in a binding site [54,55]. Moreover, a strong interaction may restrict protein movement, thereby affecting its biological activity [56]. Therefore, PCA was carried out to investigate the collective motion of unbound and bound drugs (ledipasvir, remdesivir, and paritaprevir) in the MD trajectories. In this dimensionality reduction method, a projection of two principal components, PC1 and PC2, is calculated by diagonalizing the covariance matrix of eigenvectors to describe the subspace in which maximum protein dynamics occur. Furthermore, by PCA, the MD trajectories of all the protein–ligand complexes and of the apo-protein were examined to evaluate the conformational and structural changes upon ligand binding. The dynamic motions of the protein determined by PCA are depicted in Figure 4. The unbound protein (apo-RdRp) clearly possesses fewer stable clusters compared to the RdRp–drug complexes, particularly in PC2. In the RdRp–drug complexes, a greater number of stable clusters in some protein regions were identified, indicating a reduction in the overall collective motion because the drug binding effectively limits the protein backbone movements in certain regions. The conformational space covered in the case of the ledipasvir and remdesivir complexes proved to be broader than that of the complex with paritaprevir. These results mean that upon ligand binding, the overall conformation of the RdRp protein changes, which may alter the desired protein functionality.

### 3.6. Protein Folding Dynamics Exploration

**Figure 5:**
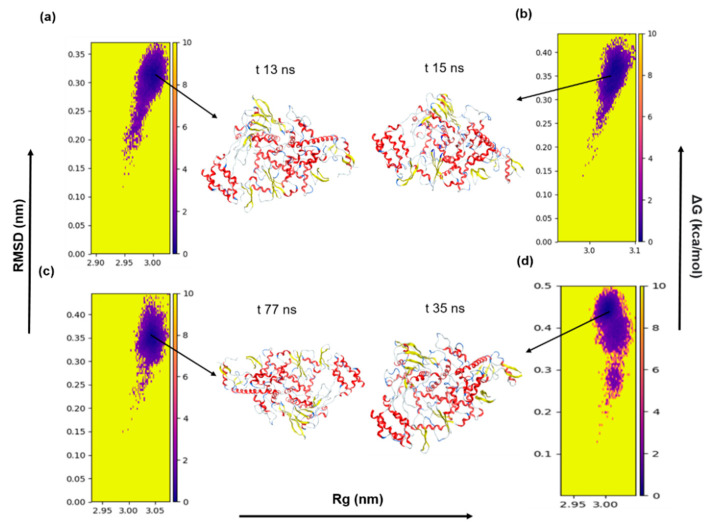
The FEL of the first two principal components as a function of the radius of gyration (Rg) and root mean square deviation (RMSD). (**a**) The apo-RdRp protein; (**b**) the RdRp protein–remdesivir complex; (**c**) the RdRp protein–ledipasvir complex; and (**d**) the RdRp protein–paritaprevir complex. Snapshots were extracted from minimum energy wells.

The landscapes of energy minima of both apo-RdRp and drug-bound RdRp were visualized using the FEL against two principal components, PC1 (RMSD) and PC2 (R_g_), which showed ΔG values between 0 and 10 kJ/mol (Figure 5). The stability of the protein is represented by the darkest and centralized blue regions of the FEL. This region in the plot denotes the energy minima of different conformations and represents the stability of the protein complex. As illustrated in Figure 5a–d, the lowest-energy state of the FEL of apo-RdRp is achieved after 13 ns, whereas the RdRp in complex with remdesivir, ledipasvir, or paritaprevir achieves stability at 15, 77, and 35 ns, respectively. Moreover, the low-energy zones of the apo form and of RdRp in complex with remdesivir or ledipasvir are larger than those of the paritaprevir complex. This finding suggests that the RdRp–paritaprevir complex goes through a comparatively lengthy transition state to achieve an equilibrium. Nonetheless, overall, the selected drugs (remdesivir, ledipasvir, and paritaprevir) have a potential to cause the RdRp protein to enter a local energy minimum state.

### 3.7. Time-Correlated Protein Domain Motions and Molecular Flexibility

**Figure 6:**
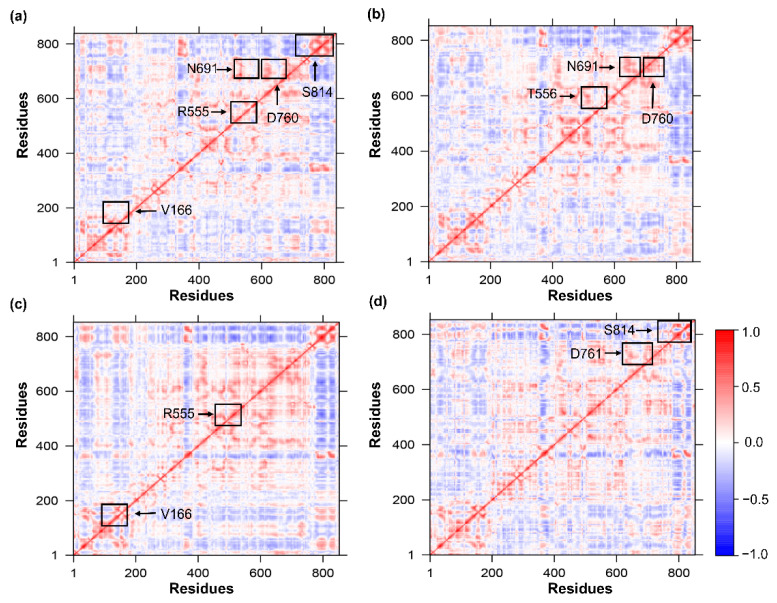
The DCCMs. The DCCM for (**a**) the apo-RdRp form and for the RdRp complex with (**b**) remdesivir, (**c**) ledipasvir, (**d**) paritaprevir. The color gradients represent a positive (red) and negative (blue) correlation.

The DCCM is a 3D matrix that graphically depicts time-correlated information on correlations among a protein’s amino acid residues. The most common method used to analyze residues based on time-correlated data is visual pattern recognition. In this regard, DCCM analysis of Cα atoms was performed throughout the simulations to probe the dynamics of apo-RdRp and of RdRp in complex with a drug (remdesivir, ledipasvir, or paritaprevir), as illustrated in Figure 6. The residues with highly positively correlated motions are shown as red regions, while highly inversely correlated motions are blue regions. According to Figure 6a, the active-site residues in the apo form undergo stronger correlated motions. In the RdRp–remdesivir complex, the active-site interacting residues (T556, N691, and D760) showed a positive correlation, just as in the apo-form. Similarly, interacting residues V166 and R555 in the ledipasvir complex and D761 and S814 in the paritaprevir complex also manifested strong correlations. These results uncovered the presence of stronger cross-correlation dynamics between residues in the RdRp–drug complexes (Figure 6b–d), suggesting strong interactions and better stability. As documented in this study, residues in these regions also have similar RMSF values in both the apo form and RdRp bound with the selected drugs. Thus, it can be concluded that the pattern of correlation observed here improves overall system stability.

### 3.8. Binding Free Energy Calculations

**Figure 7:**
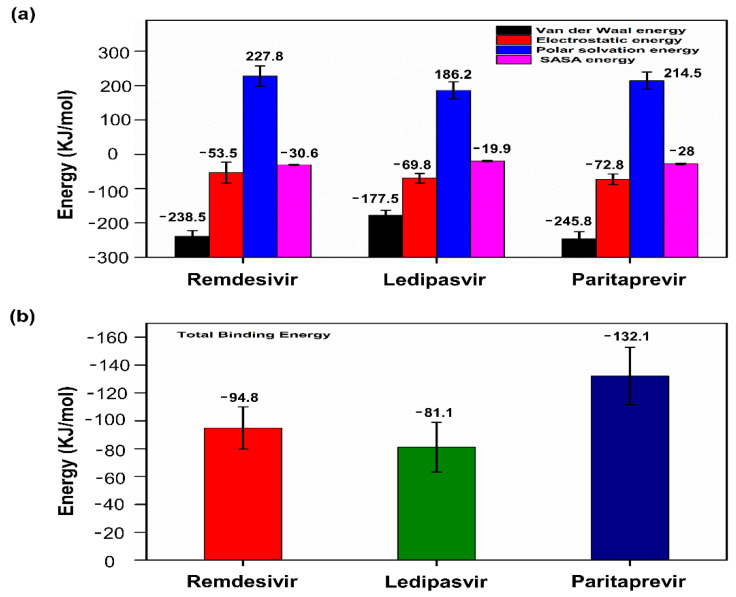
MM-PBSA binding free energy calculation. (**a**) Representative contributions of each energy component for RdRp with the selected drugs (remdesivir, ledipasvir, and paritaprevir). (**b**) The total binding free energy for RdRp in complex with the selected drugs.

The MM-PBSA method was employed to calculate the binding free energy from the trajectory obtained during MD simulation. For each protein–ligand complex, binding free energy (∆G_bind_), van der Waals energy, electrostatic energy, and polar solvation energy were calculated as shown in Figure 7a. It has been observed in previous studies that binding free energy values lower than −30 kJ/mol can be taken for binding, but lower binding free energy values are considered to be more favorable for the interaction [57,58]. In the current study, RdRp-drugs (remdesivir, ledipasvir, and paritaprevir) showed acceptable binding free energy values as shown in Figure 7b. The cumulative binding energy contributed for selected drugs remdesivir, ledipasvir, and paritaprevir were −94.877, −81.18, and −132.108 kJ/mol respectively. In all three complexes, the van der Waals interactions significantly contributes in the binding energy. Overall, our analysis established that the selected drugs have the potential to bind tightly to the SARS-CoV2 RdRp protein.

### 3.9. In Vitro Antiviral Activity

**Figure 8:**
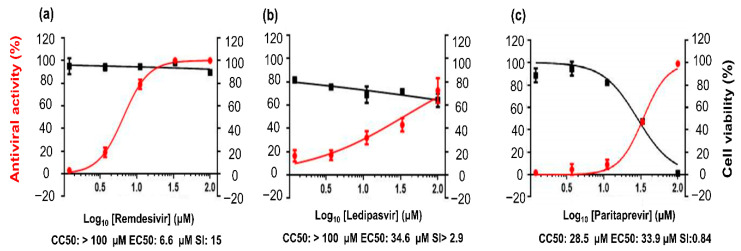
In vitro evaluation of antiviral activity of the drug candidates against SARS-CoV-2. (**a**–**c**) The right vertical axis represents cell viability, and the left vertical axis denotes antiviral activity against SARS-CoV-2.

To determine the effectiveness of the selected FDA-approved drugs against the replication of highly pathogenic SARS-CoV-2, the antiviral activities of the three drugs were evaluated next. In this assay, (a) remdesivir, an adenosine analogue that has been evaluated before the 2018 Kivu Ebola epidemic [59]; (b) ledipasvir, an orally administered NS5A inhibitor used against hepatitis C virus (HCV) [60]; and (c) paritaprevir, a 3/4A protease inhibitor prescribed against HCV infection [61] were evaluated against SARS-CoV-2. Here, we performed antiviral assays on the Vero E6 cell line, and remdesivir served as a positive control according to the observed antiviral activity. We assessed virus replication in the cultured cells exposed to various drug doses (100, 33, 11, 3.7, and 1.2 μM). Judging by the results, 50% cytotoxicity concentration (CC_50_) of remdesivir was >100 μM, EC_50_ was 6.6 μM, and the selectivity index (SI) was >15 (Figure 8a). By contrast, ledipasvir manifested antiviral activity with an EC_50_ of 34.6 μM, and CC_50_ was >100 μM with SI > 2.9 (Figure 8b). Paritaprevir was found to have an antiviral activity with EC_50_ of 33.9 μM, and CC_50_ was 28.5 μM with SI = 0.84 (Figure 8c). Although remdesivir showed a higher effective drug concentration than ledipasvir did, its SI remains of interest.

Taken together, these results mean that remdesivir has a more potent in vitro antiviral activity than ledipasvir does at various doses for the control of viral replication within 24–48 h in our assay system. We can hypothesize that the mechanism is the inhibition of the replicase complex of the virus. Unfortunately, paritaprevir does not have any useful antiviral activity against SARS-CoV-2. Remdesivir is effective against SARS-CoV-2 but has adverse effects [62]. Furthermore, it has a half-life of 0.4 h in nonhuman primates, and for this reason, it offers human angiotensin-converting enzyme 2 (ACE2) inhibition of shorter duration. By contrast, the nucleoside triphosphate metabolite of remdesivir has a half-life of 14 h in nonhuman primates and approximately 20 h in humans [63]. On the other hand, ledipasvir is not extensively metabolized, and its median terminal half-life is 47 h [64]. Due to its longer half-life, ledipasvir shows promise for prolonged action on extracellular human ACE2 as a target. Similarly, the intracellular concentration effective against RdRp in host cells is apparent from a mechanistic insight into known antiviral activity against HCV. Additionally, ledipasvir is a highly protein-bound drug (>99.8%), implying better free-drug concentration at the target site, and therefore should be suitable for an effective therapeutic intervention. Consequently, a multitarget treatment strategy involving a synergistic combination of drugs with different mechanisms of action may offer more robust practical treatment of COVID-19.

## 4. Conclusions

The present study identified suitable FDA-approved drugs for the inhibition of the SARS-CoV-2 RdRp enzyme. In this regard, a structure-based virtual screening of FDA-approved antiviral drugs was implemented to find lead molecules on the basis of the interaction with the RdRp active site. MD simulations were performed for 100 ns to investigate the dynamic behavior of protein–ligand interactions, revealing that the protein–ligand complex maintains a stable conformation with lower protein–ligand interaction energy. The top hits were then confirmed by an in vitro assay to validate the efficacy of the selected drugs experimentally against SARS-CoV-2 replication. In this context, remdesivir and ledipasvir exerted antiviral action against the virus, and unexpectedly, the antiviral activity of our selected drugs was recently identified in another cell-based screening assay against SARS-CoV-2 [65,66]. This evidence may guide the selection of downstream experiments in further studies and may be a starting point for further confirmation of the selected drugs in a synergistic combination for use in a biologically relevant and more complex preclinical model of COVID-19.

## Figures and Tables

**Table 1:** Docking scores and hydrogen-bonding interactions with important binding-site residues.

Drug Name	Hydrogen Bonds	Bond Length (Å)	Force Field	MOE Docking Score (kcal/mol)
Ledipasvir	R555	2.1 and 2.6	CHARMM36	−8.9
	V166	2.2		
Remdesivir	T556	2.1 and 2.4	CHARMM36	−8.3
	D760	2.3 and 2.5		
	N691	2.5		
Paritaprevir	D761	2.1	CHARMM36	−9.4
	S814	2.0		

## References

[1] Fehr A.R., Perlman S. (2015). Coronaviruses: An overview of their replication and pathogenesis. Coronaviruses: Methods and Protocols.

[2] Ye Z.W., Yuan S., Yuen K.S., Fung S.Y., Chan C.P., Jin D.Y. (2020). Zoonotic origins of human coronaviruses. Int. J. Biol. Sci..

[3] Aftab S.O., Ghouri M.Z., Masood M.U., Haider Z., Khan Z., Ahmad A., Munawar N. (2020). Analysis of SARS-CoV-2 RNA-dependent RNA polymerase as a potential therapeutic drug target using a computational approach. J. Transl. Med..

[4] Su S., Wong G., Shi W., Liu J., Lai A.C.K., Zhou J., Liu W., Bi Y., Gao G.F. (2016). Epidemiology, Genetic Recombination, and Pathogenesis of Coronaviruses. Trends Microbiol..

[5] Beniac D.R., Andonov A., Grudeski E., Booth T.F. (2006). Architecture of the SARS coronavirus prefusion spike. Nat. Struct. Mol. Biol..

[6] Delmas B., Laude H. (1990). Assembly of coronavirus spike protein into trimers and its role in epitope expression. J. Virol..

[7] Nal B., Chan C., Kien F., Siu L., Tse J., Chu K., Kam J., Staropoli S., Crescenzo-Chaigne B., Escriou N. (2005). Differential maturation and subcellular localization of severe acute respiratory syndrome coronavirus surface proteins S, M and E. J. Gen. Virol..

[8] Neuman B.W., Kiss G., Kunding A.H., Bhella D., Baksh M.F., Connelly S., Droese B., Klaus J.P., Makino S., Sawicki S.G. (2011). A structural analysis of M protein in coronavirus assembly and morphology. J. Struct. Biol..

[9] Wu J., Liu W., Gong P., Gong P. (2015). A structural overview of RNA-dependent RNA polymerases from the Flaviviridae family. Int. J. Mol. Sci..

[10] Smertina E., Urakova N., Strive T., Frese M. (2019). Calicivirus RNA-dependent RNA polymerases: Evolution, structure, protein dynamics, and function. Front. Microbiol..

[11] Ago H., Adachi T., Yoshida A., Yamamoto M., Habuka N., Yatsunami K., Miyano M. (1999). Crystal structure of the RNA-dependent RNA polymerase of hepatitis C virus. Structure.

[12] Elfiky A.A. (2020). Anti-HCV, nucleotide inhibitors, repurposing against COVID-19. Life Sci..

[13] Nguyen T.M., Zhang Y., Pandolfi P.P. (2020). Virus against virus: A potential treatment for 2019-nCov (SARS-CoV-2) and other RNA viruses. Cell Res..

[14] Kruse R.L. (2020). Therapeutic strategies in an outbreak scenario to treat the novel coronavirus originating in Wuhan, China. F1000Research.

[15] Lai M.M., Baric R.S., Makino S., Keck J.G., Egbert J., Leibowitz J.L., Stohlman S.A. (1985). Recombination between nonsegmented RNA genomes of murine coronaviruses. J. Virol..

[16] Li Q., Wu J., Nie J., Li X., Huang W., Correspondence Y.W., Zhang L., Hao H., Liu S., Zhao C. (2020). The Impact of Mutations in SARS-CoV-2 Spike on Viral Infectivity and Antigenicity. Cell.

[17] Xu X., Liu Y., Weiss S., Arnold E., Sarafianos S.G., Ding J. (2003). Molecular model of SARS coronavirus polymerase: Implications for biochemical functions and drug design. Nucleic Acids Res..

[18] Hansen J.L., Long A.M., Schultz S.C. (1997). Structure of the RNA-dependent RNA polymerase of poliovirus. Structure.

[19] Bressanelli S., Tomei L., Roussel A., Incitti I., Vitale R.L., Mathieu M., De Francesco R., Rey F.A. (1999). Crystal structure of the RNA-dependent RNA polymerase of hepatitis C virus. Proc. Natl. Acad. Sci. USA.

[20] Kinsella E., Martin S.G., Grolla A., Czub M., Feldmann H., Flick R. (2004). Sequence determination of the Crimean-Congo hemorrhagic fever virus L segment. Virology.

[21] Liang B., Li Z., Jenni S., Rahmeh A.A., Morin B.M., Grant T., Grigorieff N., Harrison S.C., Whelan S.P.J. (2015). Structure of the L Protein of Vesicular Stomatitis Virus from Electron Cryomicroscopy. Cell.

[22] Elfiky A.A. (2016). Zika viral polymerase inhibition using anti-HCV drugs both in market and under clinical trials. J. Med. Virol..

[23] Elfiky A.A., Ismail A. (2019). Molecular dynamics and docking reveal the potency of novel GTP derivatives against RNA dependent RNA polymerase of genotype 4a HCV. Life Sci..

[24] Yang P.L., Gao M., Lin K., Liu Q., Villareal V.A. (2011). Anti-HCV drugs in the pipeline. Curr. Opin. Virol..

[25] Gao Y., Yan L., Huang Y., Liu F., Zhao Y., Cao L., Wang T., Sun Q., Ming Z., Zhang L. (2020). Structure of the RNA-dependent RNA polymerase from COVID-19 virus. Science.

[26] (2021). Molecular Operating Environment (MOE).

[27] De Clercq E., Li G. (2016). Approved antiviral drugs over the past 50 years. Clin. Microbiol. Rev..

[28] Chaudhuri S., Symons J.A., Deval J. (2018). Innovation and trends in the development and approval of antiviral medicines: 1987–2017 and beyond. Antivir. Res..

[29] Merck Molecular Force Field (MMFF94)—CHARMM v35b1 Documentation. http://www.charmm-gui.org/charmmdoc/mmff.html.

[30] Kaminski G., Jorgensen W.L. (1996). Performance of the AMBER94, MMFF94, and OPLS-AA force fields for modeling organic liquids. J. Phys. Chem..

[31] (2015). LLC The {PyMOL} Molecular Graphics System.

[32] Yu W., He X., Vanommeslaeghe K., MacKerell A.D. (2012). Extension of the CHARMM general force field to sulfonyl-containing compounds and its utility in biomolecular simulations. J. Comput. Chem..

[33] Huang J., Mackerell A.D. (2013). CHARMM36 all-atom additive protein force field: Validation based on comparison to NMR data. J. Comput. Chem..

[34] Bussi G., Donadio D., Parrinello M. (2007). Canonical sampling through velocity rescaling. J. Chem. Phys..

[35] Essmann U., Perera L., Berkowitz M.L., Darden T., Lee H., Pedersen L.G. (1995). A smooth particle mesh Ewald method. J. Chem. Phys..

[36] Kutzner C., Páll S., Fechner M., Esztermann A., De Groot B.L., Grubmüller H. (2015). Best bang for your buck: GPU nodes for GROMACS biomolecular simulations. J. Comput. Chem..

[37] Bahar I., Atilgan A.R., Demirel M.C., Erman B. (1998). Vibrational dynamics of folded proteins: Significance of slow and fast motions in relation to function and stability. Phys. Rev. Lett..

[38] Pronk S., Páll S., Schulz R., Larsson P., Bjelkmar P., Apostolov R., Shirts M.R., Smith J.C., Kasson P.M., Van Der Spoel D. (2013). GROMACS 4.5: A high-throughput and highly parallel open source molecular simulation toolkit. Bioinformatics.

[39] Seeber M., Cecchini M., Rao F., Settanni G., Caflisch A. (2007). Wordom: A program for efficient analysis of molecular dynamics simulations. Bioinformatics.

[40] Dash R., Ali M.C., Dash N., Azad M.A.K., Hosen S.M.Z., Hannan M.A., Moon I.S. (2019). Structural and Dynamic Characterizations Highlight the Deleterious Role of SULT1A1 R213H Polymorphism in Substrate Binding. Int. J. Mol. Sci..

[41] Maisuradze G.G., Liwo A., Scheraga H.A. (2010). Relation between free energy landscapes of proteins and dynamics. J. Chem. Theory Comput..

[42] Wan H., Hu J., Li K., Tian X., Chang S. (2013). Molecular Dynamics Simulations of DNA-Free and DNA-Bound TAL Effectors. PLoS ONE.

[43] Frauenfelder H., Sligar S.G., Wolynes P.G. (1991). The energy landscapes and motions of proteins. Science.

[44] Grant B.J., Rodrigues A.P.C., ElSawy K.M., McCammon J.A., Caves L.S.D. (2006). Bio3d: An R package for the comparative analysis of protein structures. Bioinformatics.

[45] Upadhyay S.K. (2014). Dynamics of Gal80p in the Gal80p-Gal3p complex differ significantly from the dynamics in the Gal80p-Gal1p complex: Implications for the higher specificity of Gal3p. Mol. Biosyst..

[46] Chillemi G., D’Annessa I., Fiorani P., Losasso C., Benedetti P., Desideri A. (2008). Thr729 in human topoisomerase I modulates anti-cancer drug resistance by altering protein domain communications as suggested by molecular dynamics simulations. Nucleic Acids Res..

[47] Kumari R., Kumar R., Lynn A. (2014). G-mmpbsa -A GROMACS tool for high-throughput MM-PBSA calculations. J. Chem. Inf. Model..

[48] Hou T., Wang J., Li Y., Wang W. (2011). Assessing the performance of the molecular mechanics/Poisson Boltzmann surface area and molecular mechanics/generalized Born surface area methods. II. the accuracy of ranking poses generated from docking. J. Comput. Chem..

[49] Homeyer N., Gohlke H. (2012). Free energy calculations by the Molecular Mechanics Poisson-Boltzmann Surface Area method. Mol. Inform..

[50] Miller B.R., McGee T.D., Swails J.M., Homeyer N., Gohlke H., Roitberg A.E. (2012). MMPBSA.py: An efficient program for end-state free energy calculations. J. Chem. Theory Comput..

[51] Doublié S., Ellenberger T. (1998). The mechanism of action of T7 DNA polymerase. Curr. Opin. Struct. Biol..

[52] Koulgi S., Jani V., Uppuladinne M.V.N., Sonavane U., Joshi R. (2020). Remdesivir-bound and ligand-free simulations reveal the probable mechanism of inhibiting the RNA dependent RNA polymerase of severe acute respiratory syndrome coronavirus 2. RSC Adv..

[53] Boehr D.D., Nussinov R., Wright P.E. (2009). The role of dynamic conformational ensembles in biomolecular recognition. Nat. Chem. Biol..

[54] Karshikoff A., Nilsson L., Ladenstein R. (2015). Rigidity versus flexibility: The dilemma of understanding protein thermal stability. FEBS J..

[55] Hinsen K. (2008). Structural flexibility in proteins: Impact of the crystal environment. Bioinformatics.

[56] Du X., Li Y., Xia Y.L., Ai S.M., Liang J., Sang P., Ji X.L., Liu S.Q. (2016). Insights into protein–ligand interactions: Mechanisms, models, and methods. Int. J. Mol. Sci..

[57] Rifai E.A., Van Dijk M., Vermeulen N.P.E., Yanuar A., Geerke D.P. (2019). A Comparative Linear Interaction Energy and MM/PBSA Study on SIRT1-Ligand Binding Free Energy Calculation. J. Chem. Inf. Model..

[58] Poli G., Granchi C., Rizzolio F., Tuccinardi T. (2020). Application of MM-PBSA Methods in Virtual Screening. Molecules.

[59] Mulangu S., Dodd L.E., Davey R.T., Tshiani Mbaya O., Proschan M., Mukadi D., Lusakibanza Manzo M., Nzolo D., Tshomba Oloma A., Ibanda A. (2019). A Randomized, Controlled Trial of Ebola Virus Disease Therapeutics. N. Engl. J. Med..

[60] Scott L.J. (2018). Ledipasvir/Sofosbuvir: A Review in Chronic Hepatitis C. Drugs.

[61] Menon R.M., Polepally A.R., Khatri A., Awni W.M., Dutta S. (2017). Clinical Pharmacokinetics of Paritaprevir. Clin. Pharmacokinet..

[62] Wang Y., Zhang D., Du G., Du R., Zhao J., Jin Y., Fu S., Gao L., Cheng Z., Lu Q. (2020). Remdesivir in adults with severe COVID-19: A randomised, double-blind, placebo-controlled, multicentre trial. Lancet.

[63] Warren T.K., Jordan R., Lo M.K., Ray A.S., Mackman R.L., Soloveva V., Siegel D., Perron M., Bannister R., Hui H.C. (2016). Therapeutic efficacy of the small molecule GS-5734 against Ebola virus in rhesus monkeys. Nature.

[64] (2015). New drugs: Ledipasvir with sofosbuvir. Aust. Prescr..

[65] Wang M., Cao R., Zhang L., Yang X., Liu J., Xu M., Shi Z., Hu Z., Zhong W., Xiao G. (2020). Remdesivir and chloroquine effectively inhibit the recently emerged novel coronavirus (2019-nCoV) in vitro. Cell Res..

[66] Sayad B., Sobhani M., Khodarahmi R. (2020). Sofosbuvir as Repurposed Antiviral Drug Against COVID-19: Why Were We Convinced to Evaluate the Drug in a Registered/Approved Clinical Trial?. Arch. Med. Res..

